# Central Centrifugal Cicatricial Alopecia in the Adolescent Population: An Overview of Available Literature

**DOI:** 10.3390/life13041022

**Published:** 2023-04-15

**Authors:** Victoria Palmer, Manuel Valdebran

**Affiliations:** 1 King’s Medical Center, St. Catherine, Portmore JM00000, Jamaica; 2 Department of Dermatology and Dermatologic Surgery, Medical University of South Carolina, Charleston, SC 29425, USA; 3 Department of Pediatrics, Medical University of South Carolina, Charleston, SC 29425, USA

**Keywords:** central centrifugal cicatricial alopecia, scarring alopecia, pediatric, scarring hair loss, skin of colour, public health

## Abstract

Central centrifugal cicatricial alopecia (CCCA) is a lymphocytic scarring alopecia that predominantly affects women of African descent. Recent studies have demonstrated prevalence in children and adolescents, as well as Asian populations. A thorough search of Pubmed, Cochrane Database of Systematic Reviews, OVID Medline and Google Scholar was conducted using keywords such as “central centrifugal cicatricial alopecia”, “scarring hair loss”, “scarring alopecia”, “hot comb alopecia”, “pediatric” and “adolescent”. The results yielded few articles in the literature that directly addressed CCCA in the adolescent population, with three articles providing details of the presentation in the form of case series and retrospective reviews. The presentation in the adolescent population was found to be varied, ranging from asymptomatic to symptomatic and involving diffuse to patchy hair loss in only the vertex and/or frontal and parietal scalp. Genetic and environmental etiologies were found to be statistically significant, and markers of metabolic dysregulation predisposing patients to diabetes mellitus and breast cancer were also uncovered. The differential diagnosis of patients who present with hair loss in the adolescent population should therefore be broad, and a low threshold for biopsies should be adopted to confirm CCCA in suspected patients. This will have future implications for reduced morbidity and public health.

## 1. Introduction

Central Centrifugal Cicatricial Alopecia (CCCA) is a lymphocytic scarring alopecia seen more commonly in adult women of African descent, with emerging literature also suggesting a prevalence in adolescent Black and Asian populations [1,2,3]. The aetiology of CCCA is multifactorial, ranging from genetic predisposition to traumatic hairstyling practices [2]. The association with metabolic syndrome and breast cancer also promotes inquiry into relevant genetic markers and potential public health implications [3,4,5,6,7]. Therefore, a high index of suspicion in the adolescent population should be maintained as there is wide variability in the presentation of CCCA. This review aims to classify the aetiology, presentation, histopathology, diagnosis and significance of treatment of CCCA in the adolescent population.

## 2. Epidemiology

The documented incidence of scarring hair loss at the vertex, predominantly presumed to be CCCA, is 5.6% in the US [4]. Women of African descent have a higher predilection for the condition, with a female: male ratio of 3:1 [8]. Adolescent patients tend to have a late presentation, like their adult counterparts [9]. Six patients with biopsy-proven cases of CCCA had an average of 32.4 months from symptom onset to presentation [10]. This differs from a study by Imhof et al., which reports that the average time from symptom onset to the diagnosis of scarring alopecia in the pediatric population was 17.1 months [11]. However, cases of CCCA were not included in this cohort, and it should be noted that the primary target population was White or Multiracial. Epidemiological data regarding the age of symptom onset is limited; however, studies have demonstrated an average age of 14 years, ranging from 11–15 years old [10,12,13]. This is in keeping with other average ages of primary cicatricial alopecias [11]. Concurrent psychiatric co-morbidities included anxiety (22.2%) and depression (22.2%) [9,11].

## 3. Aetiology

A genetic component of CCCA has been observed: inheritance is noted to be in an autosomal dominant pattern with partial/variable penetrance in familial cases [9,10,14,15,16]. Additionally, the role of anticipation is also being studied: in a study by Eginli et al., four of five adolescent patients with CCCA had a first-degree relative, their mothers, with the condition [10]. The same finding was documented in an asymptomatic South African 11-year-old girl who had biopsy-proven CCCA, diagnosed after she was screened because of her mother’s CCCA diagnosis [12]. Scalp biopsies of CCCA have also demonstrated an upregulation of genes involved in fibroblast proliferation, collagen formation, and wound healing [9]. People of colour, i.e., Fitzpatrick phototypes IV–VI, have an increased proportion of fibroblasts relative to their White counterparts [1]. This finding may provide insight into the disparate incidence in the former population, as fibroblasts are a core component of wound healing and abnormalities with this process, manifesting in conditions such as keloids, which also prevails in People of Colour (POC) [17]. Therefore, the finding by Eginli et al. that two pediatric patients with biopsy-confirmed CCCA had previous scalp surgery may be linked to this pathogenesis [10]. Additionally, peptidyl arginine deiminase type III (PADI3), a gene involved in lipid metabolism and hair shaft formation, may be downregulated in patients with CCCA [9,15]. This decrease in expression, as well as concomitant missense and splice mutations, ultimately leads to decreased follicular development and abnormalities of the hair shaft resulting in increased hair fragility and breakage [9,15]. A disruption of pro-inflammatory and anti-inflammatory factors has also been noted to contribute to the pathogenesis. For example, matrix metalloproteinase 9 (MMP9) is a biomarker associated with dysregulated pathways of fibrosis and is upregulated in severe cases of CCCA [18]. This dysregulation is translated clinically by the low-grade inflammation seen on dermoscopy and histopathology with the subsequent progression to fibrosis [15].

Ethnic hair practices are the most debated aetiology for CCCA. While the majority of patients with CCCA have reported traumatic hairstyling practices (chemical relaxers/braids/hotcombs/weaves) at least once in their lifetime, these findings may not be causative [13,14]. Chemical relaxers have not consistently been associated with statistically significant findings [4,19,20]. Rather, bacterial scalp infections and tension-inducing hairstyles such as weaves/extensions and braids/cornrows have the most statistically significant correlation with clinically diagnosed CCCA, although this still remains a matter of debate [21,22,23]. The hypothesis that alopecia may result from tension-inducing hairstyles is consistent with the dysregulated pro-inflammatory and anti-inflammatory signals involved in the pathogenesis of CCCA; Further histopathological studies comparing the extent of inflammation between tension-inducing hairstyles and chemical relaxers would be useful in delineating a clearer association. Tension hairstyles are applied relatively frequently and involve consistent traction and subsequent inflammation for days to weeks at a time, while chemical relaxers are briefly applied to the scalp and are most frequently reapplied at 4-to-8-week intervals. The level of inflammation in tension hairstyles, though presumably milder, may therefore have a more profound cumulative effect. This is of cultural significance as hair grooming practices involving the use of heavy beads, cornrows and tight ponytails start at an early age in the African American community, highlighting the necessity of parent education.

## 4. Clinical Associations

Evidence demonstrates that CCCA may be a marker of metabolic dysregulation [7,11,15]. Roche et al. demonstrated that nonobese women with biopsy-proven CCCA had over a 3× risk of developing diabetes mellitus type 2 than their age, race and sex-matched controls ((OR (95% CI)): 3.26 (1.33–8.04); *p*-value < 0.05)) [7]. A 4.68× increase in the odds of having uterine leiomyomas compared with race-, age-, and sex-matched controls was also observed in a retrospective study at Johns Hopkins Hospital (*p* < 0.001) [24]. Additionally, Brown et al. demonstrated that in patients with biopsy-proven CCCA (159 of 742), 4.4% had a history of breast cancer compared with the 4079 controls (1.8%) (odds ratio 2.49; 95% confidence interval 1.06–4.92; *p* = 0.02) [25]. Both latter conditions are associated with an abnormal proliferation of fibrous tissue and once again demonstrate a crossover of the pathogenesis to different organ systems. The average age of diagnosis of breast cancer was 62.9 years [25]. While the types of breast cancer were not delineated in the study, an association between dysregulated PADI3, upregulated MMP9, triple-negative inflammatory breast cancer (TNIBC), and triple-negative non-inflammatory breast cancer (TN-NIBC) has been documented in the literature [5,6,23]. Triple-negative breast cancer appears more frequently in women aged 40 and younger than in older women. Black and Latina women are more predisposed to develop TNBC than white women. Women who have the gene change BRCA1 are also more likely to develop TNBC than other women [26]. Further investigation is therefore needed into the types of breast cancer diagnosed in the retrospective cross-sectional study by Brown et al., as it may help to broaden the scope of associated genetic markers to screen in the younger population [25].

## 5. Presentation

**Figure 1:**
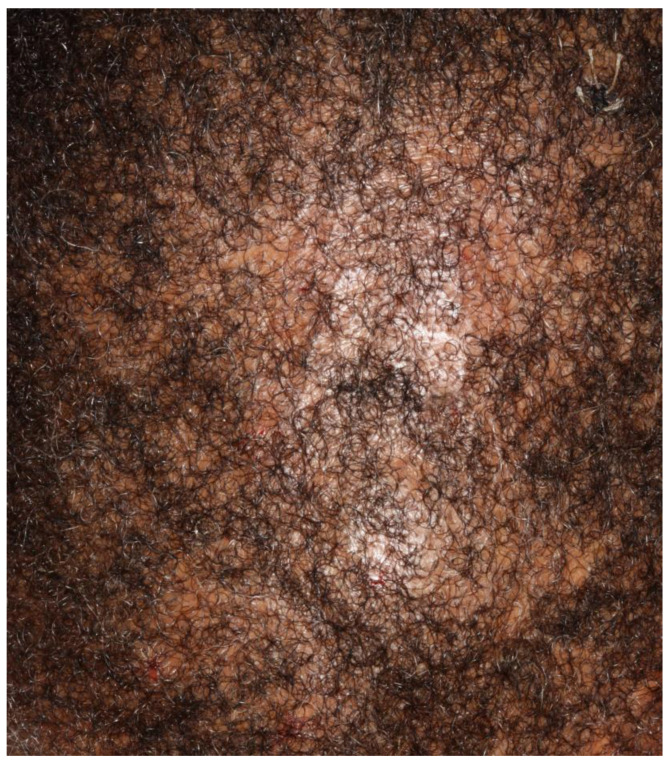
Patchy scarring alopecia with underlying erythema and erosions in male patient with biopsy-proven CCCA.

Eginli et al. analysed the scalp symptoms of six patients aged 14–19 who had symptom onset of biopsy-proven CCCA at 11–17 years old [10]. The primary clinical manifestation of the disease was scalp pruritus, scaling and tender papules and pustules (*n*: 5/6 = 83%). However, one patient was asymptomatic (*n*: 1/6 = 17%). The distribution of alopecia and associated signs were diverse: areas involved ranged from patchy hair loss confined to the vertex (*n*: 4/6 = 67%) or also involving the parietal area (*n*: 1/6 = 17%) or frontal scalp (*n*: 2/6 = 33%), diffuse thinning by the temples (*n*: 1/6 = 17%), diffuse erythema without scale (*n*: 1/6 = 17%) or diffuse scaling (*n*: 2/6 = 33%) (Figure 1).

These broad presentations are in keeping with Dlova et al.’s findings of an asymptomatic 11-year-old girl with small areas of scarring on her vertex and frontal scalp, as well as Shah et al.’s findings of an 18-year-old male with pruritus, scaling/crusting, bleeding and pustules/papules for 3 years [12,13]. Of note, the majority of these patients had no history of chemical or traumatic exposure to their hair. This reinforces the importance of genetic predisposition in the pathophysiology and the importance of maintaining a wide differential diagnosis, as seen in Table 1 below.

Although long-standing tinea capitis is a known precipitator of secondary scarring alopecia, further studies are needed to delineate the complete association between fungal/bacterial infections and CCCA. *Trichophyton tonsurans* tinea capitis infections have a predilection for Afro-American and Afro-Caribbean children in both North America and Europe, with a study in London demonstrating that 91% of *T. tonsurans* tinea capitis occurred in Afro-Caribbean children [27]. Potential causes for this finding include the practice of tight braiding, which expose the scalp’s stratum corneum to fungal spores, as well as the application of oils, which may adhere to and retain spores [27]. The tight braiding hypothesis would subsequently increase the risk of developing CCCA in those who are genetically acceptable. Therefore, it would be reasonable to screen for CCCA in Afro-American or Afro-Caribbean children who present with pruritus, scaling, hair loss and black dots on dermoscopy, especially if they have a history of tension-inducing hairstyles and a maternal family history of CCCA, as all of these findings overlap with the presentation of CCCA. In addition, *T. tonsurans* can also exist as a carrier state in asymptomatic children, so these authors propose that asymptomatic children with clinical features suggestive of CCCA also have laboratory tests done to assess for concurrent infection [27].

## 6. Histopathology and Dermoscopy

The histopathologic features of CCCA are consistent across age groups [12]. Table 2 describes the histopathological findings of CCCA in pediatric populations from studies currently available in the literature.

**Figure 2:**
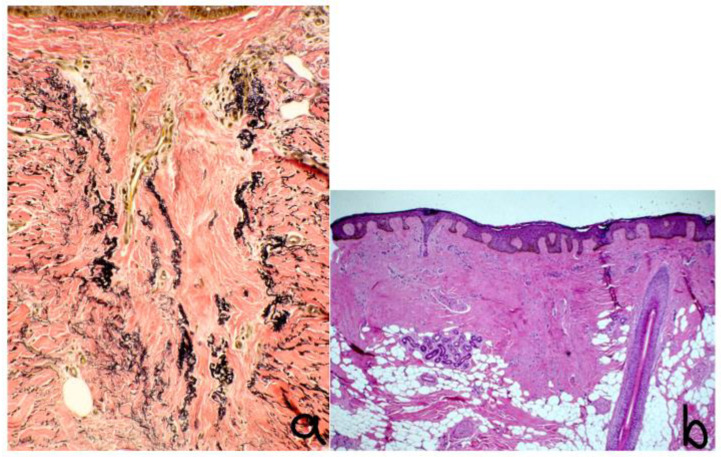
Histology: ×40 vertical sections showing (**a**) preservation and duplication of elastic sheath, (**b**) condensation of dermal collagen, broad fibrous tract with hair granuloma. Stained with hematoxylin.

**Figure 3:**
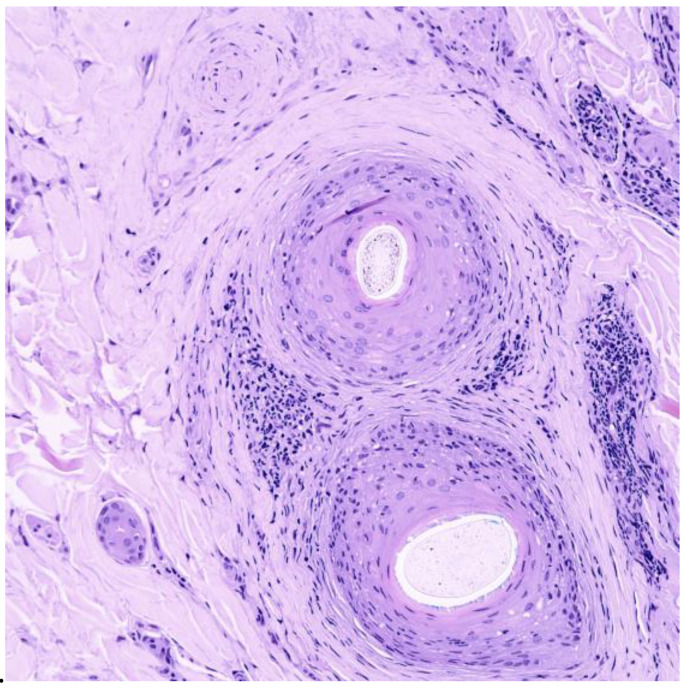
Histology ×40 horizontal section showing concentric lamellar fibroplasia and perifollicular inflammation. Stained with hematoxylin.

In the active stages of CCCA, there is follicular lichenoid inflammation; in the later stages, follicular fibrosis prevails [16]. The main diagnostic features of histopathology for CCCA are:

1. Reduced follicular density with altered architecture because of absent or reduced and miniaturised sebaceous glands [16];
2. Premature desquamation of the inner root sheath (very sensitive for CCCA, even in the absence of other findings) [16];
3. Perifollicular fibrosis and mild inflammatory infiltrate; CD3 and CD4 t-lymphocyte involvement in affected and unaffected follicles, with CD4 predominance in affected follicles and an increased CD1a:CD3 ratio [9,16] (Figure 2 and Figure 3);
4. Lamellar hyperkeratosis or parakeratosis [16];
5. Naked hair shafts in the dermis [16];
6. Follicular miniaturisation [16];
7. Increased distance between affected follicles and small blood vessel clusters (BVCs; i.e., the perifollicular mucinous fibroplasia from chronic inflammation results in diminished blood supply [9].

Dermoscopic findings include perihilar white/grey halos, loss of follicular ostia, perifollicular hyperpigmentation/erythema, polytrichia and hair shaft variability, pin-point white macules, and broken hairs resembling black dots [14,15]. Currently, there are no data correlating dermoscopy and pathology findings with disease evolution.

## 7. Management

Treatment of CCCA is notoriously challenging, and multiple therapies are often deployed, particularly in patients with advanced stages of hair loss. Early intervention is paramount to delay and/or prevent follicular burnout [21]. History includes documentation of hair and scalp symptoms, with the physician maintaining a high index suspicion as hair loss may present only as hair breakage at the vertex. The physician should also aim to confirm the diagnosis of CCCA with a biopsy to rule out other inflammatory disorders, such as lichen planopilaris (LPP) or folliculitis decalvans, which would influence subsequent management. Furthermore, documentation of hair grooming practices, gynaecological history, surgical history, medication use and dietary intake should also be obtained [9].

Treatments focus on decreasing potential triggers leading to fibrosis [9,10]. One of the first-line therapies is, therefore, behavioural modification: Although no consensus for hair styling recommendations has been reached, based on the conflicting data in the literature, it is advisable to refrain from tension-inducing hairstyles and chemical relaxers. The authors also suggest education about “protective hairstyles”. “Protective hairstyles” have been advertised by hair stylists on social media with the goal of reducing daily manipulation of the hair as well as harsh environmental exposures [28]. These hairstyles range from cornrows and bantu knots to braided lace wigs and crotchet styling [28]. However, the use of hair extensions and tugging on the hair for braiding may inadvertently apply excess tension to the scalp and exacerbate the underlying inflammation [9,10,15,28]. Parents should therefore be acutely aware of signs that their child’s hairstyle is too tight. Symptoms of excessive traction include discomfort and pruritus, and signs include visible tenting and folliculitis of the scalp [29]. If any of these signs or symptoms are observed, parents are advised to remove the hairstyle immediately [29]. In general, a braid is considered loose enough if a pencil can slip underneath the braid easily. 

**Figure 4:**
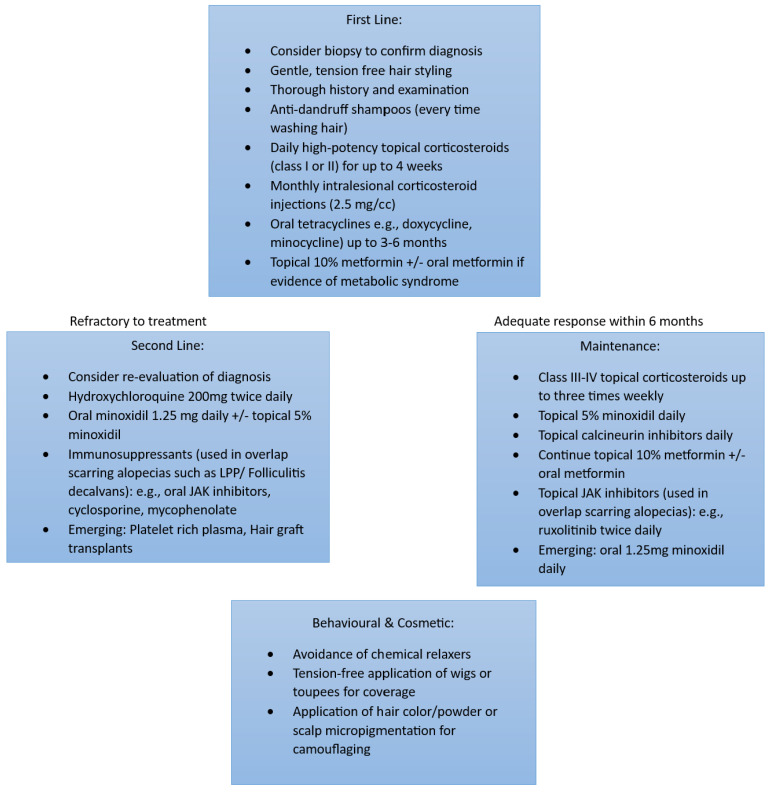
Illustrating the recommended management of CCCA. Adapted from George EA et al. [9].

Additional anti-inflammatory therapies involve the application of daily high-potency topical corticosteroids, monthly intralesional corticosteroids +/− oral anti-inflammatory antibiotics such as tetracyclines, topical minoxidil and as-needed anti-dandruff shampoos [9,10]. Topical 10% metformin and oral metformin formulations have also been prescribed based on the association of CCCA with metabolic syndrome [23,29]. A six-month trial of these first-line therapies may be utilised before considering re-evaluating the diagnosis and/or considering alternate second-line therapies (Figure 4).

For responders, results are maintained with mild–moderate potency topical corticosteroids, topical calcineurin inhibitors and topical/oral minoxidil. For refractory cases, if there was a significant infiltrate on initial biopsy, consider another type of cicatricial alopecia, such as LPP or folliculitis decalvans, in the differential diagnosis. Other strategies also include re-biopsy, reviewing the initial biopsy or seeking a second opinion. Second-line therapies include hydroxychloroquine, oral immunomodulators and oral/topical minoxidil. Immunosuppressants such as cyclosporine, mycophenolate and oral/topical JAK inhibitors are generally reserved for overlapping cases of scarring alopecia such as the aforementioned LPP or folliculitis decalvans. Surgery may be directed at end-stage biopsy-proven CCCA, in which there is no active inflammation for at least 9 to 12 months. Emerging therapies such as platelet-rich plasma injections and hair graft transplants have been described [9,14]. 

## 8. Recommendations

The postulated genetic anticipation promotes the recommendation that entire families should be screened if anyone presents with CCCA. This could be a method of decreasing the morbidity, such as concomitant anxiety and depression, associated with the disease [11]. The association between breast cancer and diabetes mellitus type II are also additional reasons to implement screening in these populations. Further studies should demonstrate the type of breast cancer associated with CCCA, as triple-negative breast cancer is associated with dysregulated PADI3 and MMP9 [5]. Women with the BRCA1 mutation are more likely to develop TNIBC; therefore, screening of this genetic marker in adolescent women with CCCA could potentially be of clinical utility [26].

## 9. Conclusions

Few studies are available in the literature outlining the presentation, management and prognosis of adolescent patients with CCCA. Awareness of CCCA in this population is paramount as the clinical presentation largely overlaps with tinea capitis scalp infections, which similarly prevail in Afro-American and Afro-Caribbean populations. A low threshold for screening and obtaining a biopsy in areas with follicular dropout should be used, particularly if first-degree relatives have concurrent hair loss. The public health implications of screening for BRCA1 mutations and diabetes mellitus type 2 should also be further researched.

## Figures and Tables

**Table 1:** Differential diagnoses of primary and secondary scarring alopecias in the pediatric population [11].

Type of Scarring Alopecia	Symptoms	Distribution of Scalp Alopecia
Central Centrifugal Cicatricial Alopecia	Scalp pruritus, scaling, tender papules or pustules, asymptomatic	Vertex, frontal, parietal or patchy distribution
Folliculitis Decalvans	Follicular hyperkeratosis, tufting, pustules, scalp induration and atrophy, mild pain or discomfort	Vertex or patchy distribution
Lichen Planopilaris (LPP)	Single or multiple plaques with itching, stinging or burning	Classic LPP: Confluent random plaques FFA: Primarily frontotemporal involvement Lasseur-Graham-Little-Picardi: Patchy distribution
Aplasia Cutis Congenita	Asymptomatic; characterised by absence of skin	Primarily vertex involvement
Tina Capitis	Scalp pruritus, scaling, tender papules or pustules, asymptomatic	Patchy distribution
Morphea (Localised Scleroderma)	Erythematous patches or plaques with itching, tenderness or asymptomatic	Variable
Discoid Lupus Erythematosus	Scaling, erythematous annular plaques, asymptomatic	Variable
Dissecting Cellulitis	Painful nodules and abscesses, scaling	Variable
Keratosis Follicularis Spinulosa Decalvans	Keratotic follicular papules, atrophic depression	Variable

**Table 2:** Histopathological findings of CCCA in various pediatric populations.

Study	Age at Time of Biopsy (Age at Onset of Symptoms)	Histopathologic Findings of Biopsy-Proven-CCCA
Dlova et al. [12]	11 (No Data)	Perifollicular and perivascular lymphoid cell infiltrate with eccentric thinning of the follicular epithelium with concentric lamellar fibroplasia
Eginli et al. [10]	14 (11) 15 (13) 16 (12) 17 (15) 19 (16) 19 (17)	Concentric perifollicular fibrosis and lymphocytes at the level of the infundibulum and isthmus
Shah et al. [13]	18 (15–16)	Scarring, perifollicular fibrosis, fibrotic tracts, inflammatory infiltrate, few follicles

## Data Availability

Data sharing is not applicable to this article as no datasets were generated or analysed during the current study.
